# Molecular Actions of Cyclophosphamide (CPA) in the Ovaries of Rats with Mammary Neoplasia

**DOI:** 10.2147/CMAR.S557751

**Published:** 2026-01-09

**Authors:** Anna Nynca, Sylwia Swigonska, Tomasz Molcan, Brian K Petroff, Renata E Ciereszko

**Affiliations:** 1 Department of Animal Anatomy and Physiology, Faculty of Biology and Biotechnology, University of Warmia and Mazury in Olsztyn, Olsztyn, Poland; 2 Department of Biochemistry, Faculty of Biology and Biotechnology, University of Warmia and Mazury in Olsztyn, Olsztyn, Poland; 3 Molecular Biology Laboratory, Institute of Animal Reproduction and Food Research, Polish Academy of Sciences, Olsztyn, Poland; 4 Department of Pathobiology and Diagnostic Investigation, Michigan State University, East Lansing, MI, USA

**Keywords:** breast cancer, cyclophosphamide, ovary, tumor-bearing rats, transcriptome, proteome

## Abstract

**Introduction:**

Women diagnosed with cancer often undergo aggressive chemotherapy that can impair fertility and lead to long-term ovarian damage, significantly affecting their quality of life. Cyclophosphamide (CPA), a chemotherapeutic agent known for its gonadotoxic effects, has been shown to reduce ovarian follicle reserves, thereby contributing to the development of primary ovarian insufficiency in both humans and animal models. This study sought to identify the molecules and intracellular signaling pathways associated with CPA’s effects on ovarian tissue of rats bearing mammary tumors.

**Methods:**

To address the objective of the study, transcriptomic (RNA-Seq) and proteomic (2D-DIGE/MS) methodologies were applied. The study was conducted on rats with N-methyl-N-nitrosourea (MNU)-induced mammary neoplasia, randomly assigned to control or cyclophosphamide (CPA)-treated groups. CPA was administered intraperitoneally (50 mg/kg on day 3, then 10 mg/kg weekly until day 31). Animals were euthanized on day 34, and ovaries were collected for RNA-Seq and 2D-DIGE/MS analyses.

**Results:**

Our results demonstrated that the crucial mechanism of CPA action during follicular depletion in the ovary may be linked to CPA-induced immune cell responses. Moreover, we found that CPA may trigger apoptosis or ferroptosis of follicular cells, ultimately leading to ovarian dysfunction.

**Conclusion:**

The obtained results highlight the importance of mechanisms contributing to ovarian toxicity from cancer chemotherapy, paving the way for developing targeted strategies for ovarian protection. Further functional experiments are needed to identify substances that could effectively preserve the fertility of female cancer survivors.

## Graphical Abstract

**Figure:**
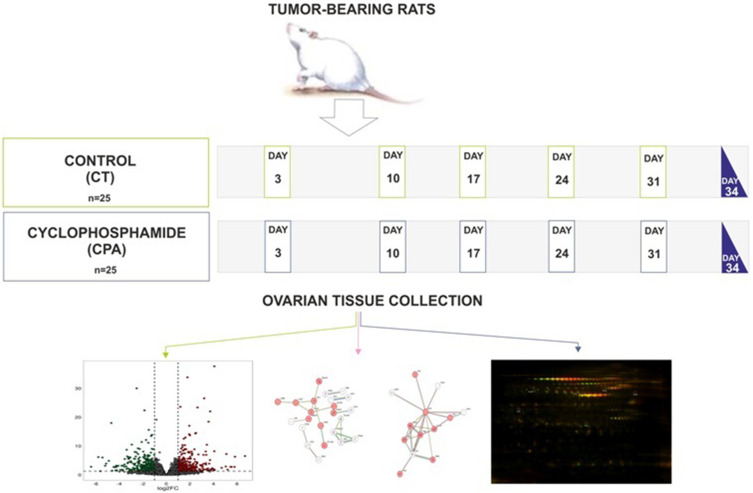

## Introduction

Intensive chemotherapy regimens, such as those including the alkylating agent cyclophosphamide (CPA), are routinely administered to women with cancer to reduce the risk of recurrence and metastatic progression. However, these treatments are frequently associated with ovarian toxicity, which has profound implications for fertility and the overall quality of life of long-term survivors. Ovarian follicles are particularly sensitive to the gonadotoxic effects of CPA and other chemotherapeutic agents.^1^ Numerous research teams, including ours, have demonstrated that CPA diminishes the ovarian follicle count, causing primary ovarian insufficiency in both human and animal models.^2–4^ Even when ovarian follicles survive exposure to gonadotoxic agents, such treatment may induce genomic alterations in the remaining follicles, potentially leading to intergenerational consequences.^5^

Proposed mechanisms underlying chemotherapy-induced follicle depletion include: 1) direct DNA damage leading to apoptotic cell death, 2) indirect effects via increased primordial follicle activation and 3) indirect effects through stromal and microvascular damage.^3^,^6^ However, there have been varying opinions on these mechanisms, and newly discovered intracellular actions of CPA that may lead to ovarian damage are still being evaluated.^6–8^ For example, CPA has been reported to cause cellular senescence in ovarian granulosa cells, which may contribute to ovarian injury.^9^ Furthermore, CPA treatment may trigger immunogenic cell death (ICD), a regulated form of cell death characterized by the surface exposure, secretion, or release of damage-associated molecular patterns (DAMPs), which function as potent immunostimulatory signals.^10^ Recently, CPA-induced ovarian dysfunction was also linked to ferroptosis-mediated cell death of granulosa cells,^7^ a process driven by iron-dependent oxidative injury that culminates in plasma membrane rupture and subsequent DAMPs release. These recent discoveries regarding the intracellular actions of CPA complicate our existing understanding of this drug’s effects, but provide a basis for discovering new pathways to limit CPA-induced ovarian dysfunction.

The loss or impairment of ovarian function as a side effect of cancer chemotherapy has been extensively studied in cancer patients and animal models.^6^,^11^ The extent of ovarian damage induced by chemotherapy is influenced by various factors, including the specific drug(s) and dosage, the developmental stage of the follicle at the time of treatment, the patient’s age and the duration of the therapy. There is an urgent need to develop a variety of options for ovarian fertility preservation and in recent decades, scientific research has focused on developing improved solutions. Most commonly used strategies, such as oocyte, embryo and ovarian tissue cryopreservation, are not universally applicable to all women and come with considerable limitations.^12^ Consequently, there is growing interest in protective pharmacological methods aimed at preserving the ovarian reserve by reducing follicle depletion through targeting the mechanisms involved in chemotherapy-induced follicle loss.

Previously, we demonstrated that tamoxifen – a selective estrogen receptor modulator commonly employed for breast cancer adjuvant therapy or chemoprevention – reduced ovarian follicle loss in a rat mammary cancer model without compromising cancer treatment with CPA.^4^ We found that this protective effect may result from tamoxifen-induced changes in the expression of genes and proteins related to apoptosis, DNA repair pathways, cell adhesion and extracellular matrix remodeling. Several other substances are also being examined for their potential to inhibit key processes involved in follicle depletion, either by limiting oocyte growth and follicular cell proliferation during the transition to growing follicles, or by inhibiting various cell death mechanisms.^3^

Finding effective strategies to prevent CPA-mediated ovarian damage requires a detailed understanding of the molecular pathways that govern ovarian follicular and stromal cell death following chemotherapy. This understanding will enable the design of targeted fertility preservation approaches in the future. Therefore, in the present study, we utilized transcriptomic and proteomic techniques to comprehensively investigate the molecular pathways that may underly CPA-induced ovarian toxicity. Moreover, unlike many previous animal experiments, the present study was conducted on rats with mammary tumors, which more reliably reflects the mechanisms occurring in breast cancer patients treated with CPA.

## Materials and Methods

### Animals and Treatments

The study protocol received approval from the Local Ethics Committee for Animal Experiments in Olsztyn, Poland (decision No. 78/2017/WNP). All experiments were performed in accordance with regulations of polish act on the protection of animals used for scientific or educational purposes. The current study is presented in compliance with the ARRIVE guidelines (Animal Research: Reporting of In Vivo Experiments). Female Wistar rats (6 weeks old) were housed under controlled conditions (22°C, 60% humidity, 12 h light:12 h dark cycle) at the Center of Experimental Medicine (Bialystok, Poland), with *ad libitum* access to food and water. The study was conducted on rats with N-methyl-N-nitrosourea (MNU)-induced mammary neoplasia.^4^ Animals were randomly assigned to two groups: 1) control (CT) and 2) cyclophosphamide (CPA)-treated. Rats in the CPA group received an intraperitoneal injection of 50 mg/kg body weight of CPA (Sigma, St. Louis, USA; in 0.9% NaCl) on day 3, followed by weekly injections of 10 mg/kg body weight on days 10, 17, 24, and 31, administered at approximately 10:00 am.^13^ Control rats received placebo injections (0.9% NaCl) at corresponding to CPA times. All rats were euthanized on day 34 of the experiment. Anesthesia was induced by administering 4% isoflurane in medical oxygen, under the supervision of a veterinarian. Ovaries were collected, snap frozen in liquid nitrogen and stored in −80°C for subsequent analyses, including RNA sequencing (RNA-Seq), two-dimensional difference gel electrophoresis (2D-DIGE), and mass spectrometry (MS).

### Isolation of Total RNA and RNA Sequencing

Total RNA was isolated from the ovaries (n=4 rats per group) with the use of peqGold TriFast reagent. RNA concentration and purity were assessed spectrophotometrically (NanoVue Plus, GE Healthcare, Little Chalfont, UK). In order to assess the integrity of total RNA, samples were run on 2100 Bioanalyzer (Agilent Technologies, Santa Clara, CA, USA). Only high-quality RNA samples with RIN (RNA integrity number; 28S/18S ratio) values ≥ 8.0 were used to construct cDNA libraries. TruSeq stranded mRNA Sample Preparation Kit (Illumina, San Diego, USA) was employed to prepare the libraries. After reverse transcription, cDNA samples were randomly fragmented, followed by ligation of 5’ and 3’ adapters. Adapter-ligated fragments were amplified by PCR, and the resulting cDNA libraries were loaded onto flow cells, where fragments were captured on a surface coated with oligonucleotides complementary to the library adapters. Each fragment was then amplified into a distinct clonal cluster via bridge amplification. Upon completion of cluster generation, the cDNA templates were prepared for sequencing. High-throughput sequencing was performed on an Illumina NovaSeq 6000 platform using a 100 bp paired-end configuration (Macrogen, Seoul, Republic of Korea).

### Identification of lncRNA

Raw read quality was assessed using FASTQC (https://www.bioinformatics.babraham.ac.uk/projects/fastqc/). Low-quality reads and adapter sequences were removed with Trimmomatic (version 0.39; doi: 10.1093/bioinformatics/btu170). Filtered reads were mapped to the rat reference genome (mRatBN7.2; Ensembl release 107) using STAR (version 2.7.10a; doi: 10.1093/bioinformatics/bts635), and mapped reads were assembled into transcripts with StringTie (version 2.2.1; doi: 10.1038/nbt.3122). Assembled transcripts were compared to the reference annotation using Gffcompare (version 0.12.6; doi: 10.12688/f1000research.23297.1). Only transcripts with class codes “x”, “o”, “i”, “u”, and “j” were retained to predict novel lncRNAs.^14^ Transcripts shorter than 200 nt or containing fewer than 2 exons were discarded to reduce potential biases. Coding potential was assessed using TransDecoder (version 5.5.0), and transcripts with open reading frames (ORFs) shorter than 300 nt (100 amino acids) were excluded. A random forest (RF) classifier was then developed to predict lncRNA transcripts. Nucleotide sequences of mRNAs and lncRNAs for human (GRCh38) and rat (mRatBN7.2) were retrieved from Ensembl (release 107). Transcripts with identical nucleotide sequences were deduplicated in both CPA-treated and control groups to avoid bias. Subsequently, features for each transcript were extracted using LncFinder 1.1.4 (doi: 10.1093/bib/bby065), and these features were used to train a RF classifier with 10-fold cross-validation using scikit-learn v1.1.2 library. Precision and recall of the RF model were evaluated on a test dataset during hyperparameter tuning. With the use of this model transcripts classified as mRNAs were excluded, while those predicted as lncRNAs were retained for further analysis. Potential lncRNAs were further searched for orthologs using BLAST (version 2.13.0; doi: 10.1186/1471-2105-10-421) against the NCBI Nucleotide Database (downloaded on 20.10.2022), with an E-value threshold set to 10^−5^. Based on BLASTn results, transcripts were classified into three categories: 1) significant matches to known lncRNA genes, 2) no significant matches, and 3) significant matches to protein-coding or non-lncRNA transcripts. Transcripts from categories 1 and 2 were retained as novel lncRNAs, while category 3 transcripts were excluded from further analysis.

### Analysis of Differentially Expressed lncRNAs (DELs) and Genes (DEGs)

Following the identification of lncRNAs, raw gene counts were obtained using featureCounts (version 2.0.3; doi: 10.1093/bioinformatics/btt656). Differentially expressed lncRNAs (DELs) and genes (DEGs), together with their adjusted P-values, were identified using R (v4.2.1) and the DESeq2 package (v1.36.0; doi: 10.1186/s13059-014-0550-8). Differential expression was defined as an adjusted P-value ≤ 0.05 and a log2 fold change (log2FC) ≥ 1.0 or ≤ −1.0. Data visualization was performed in R using the ggplot2 (v3.3.2) and ComplexHeatmap (v2.20.0; doi: 10.1093/bioinformatics/btw313) packages.

### Prediction of Cis- and Trans-Regulated lncRNA Target Genes

To evaluate cis- and trans-type interactions, Pearson’s correlation coefficients were calculated between the expression profiles of DELs and DEGs using the Hmisc package (version 4.6–0) in R. For cis-type interactions, only DEGs positioned within 20 kb of the corresponding DELs were included. P-values for lncRNA–mRNA associations were adjusted for multiple testing using the Benjamini–Hochberg procedure. Interactions were defined as significant at FDR < 0.05 with correlation coefficients of R^2^ ≥ 0.90 or R^2^ ≤ −0.90.

### Functional Enrichment Analysis

To functionally characterize the identified DEGs and the DEGs targeted by DELs, enrichment analyses were performed against the Gene Ontology (GO) and Kyoto Encyclopedia of Genes and Genomes (KEGG) databases. GO enrichment was conducted in R software using the clusterProfiler (version 4.4.4), DOSE (version 3.22.1), biomaRt (version 2.52.0), and org.Rn.eg.db (version 3.15.0) packages, while KEGG pathway analysis was performed with the clusterProfiler, DOSE, and org.Rn.eg.db packages. In both cases, enrichment was considered significant at P-adjusted < 0.05. Graphical representation of the results was obtained using ggplot2. To investigate molecular interactions, DEGs were additionally queried against the STRING v12.0 database^15^ using rat-specific data. Interactions were inferred from co-expression, text mining, and experimental evidence, with a minimum confidence score threshold of 0.4.

### Real-Time PCR

Real-time PCR was employed to validate the RNA-Seq results by assessing the expression of two selected DEGs (Bbc3 and Il12rb2) in rat ovaries collected from control and CPA-treated animals (n=4 per group). The reverse transcription (RT) reaction and real-time PCR were performed as described in previous studies.^16^,^17^ The primers and probes (Thermofisher Scientific, Waltham, MA, USA) for the specific genes are listed in Table S1.

### Protein Extraction and 2D-DIGE Analysis

Proteins were extracted from rat ovaries (n = 6 per group) using lysis buffer containing 7 M urea, 2% (w/v) CHAPS, 2% ampholytes (pH 4–7 NL; GE Healthcare, Chicago, IL, USA), 120 mM dithiothreitol, a protease inhibitor cocktail, and 0.002% bromophenol blue (Sigma Aldrich). Extraction and purification were performed as previously described.^18^ Protein concentrations were quantified before and after purification using a 2D-PAGE–adapted Bradford assay,^19^ with bovine serum albumin (BSA) dissolved in rehydration buffer (7 M urea, 2 M thiourea, 2% CHAPS, 130 mM DTT, 2% ampholytes, pH 4–7 NL) serving as a standard. Both standards and samples were acidified with 10 μL of 0.1 M HCl prior to measurement at 595 nm using an Infinite M200 multimode microplate reader (Tecan, Grödig, Austria).

Protein extracts (50 μg) from each ovary sample were solubilized in labeling buffer (30 mM Tris, 7 M urea, 2 M thiourea, 4% CHAPS, pH 8.0) and labelled with CyDye DIGE Fluor minimal dyes (GE Healthcare) at a concentration of 400 pmol dye per 50 μg protein. Labeling was carried out in the dark on ice for 30 min to prevent photobleaching. Cy2-, Cy3-, and Cy5-labelled proteins were then combined, and a dye-swap strategy was employed between control and CPA-treated groups to minimize dye bias. Proteins were separated by isoelectric focusing followed by SDS-PAGE (12.5% polyacrylamide gels) using the Ettan DALTsix electrophoresis system (GE Healthcare) at 20°C (1.5 W/gel, 16 h), as previously described.^18^ Gels were scanned on an Ettan DIGE Imager (GE Healthcare), and images were processed using SameSpots software (Totallab, Newcastle, UK). Spot intensities were normalized to the Cy2-labelled internal standard. Differentially expressed protein spots (DEPSs) were defined as those exhibiting significant changes in abundance (P < 0.05, |fold change| ≥ 1.5) between control and CPA-treated groups. Selected DEPSs were subjected to mass spectrometry (MS) for protein identification.

### Digestion of the Protein and MALDI-TOF/TOF Analysis

Gels were subsequently re-stained with Coomassie Brilliant Blue G-250 (Bio-Rad, Hercules, CA, USA), and the DEPSs from the 2D separations were carefully excised. The proteins within these spots were subjected to enzymatic digestion, and mass spectrometry analysis (MALDI-TOF/TOF) was performed following previously established protocols.^18^ Protein identifications, based on peptide mass fingerprints and ion scores, were evaluated using MASCOT software. Proteins achieving a MASCOT score above 70 (P < 0.05) were considered statistically significant.

## Results

### Impact of CPA on the Ovarian mRNA Expression Profile in Rats

**Figure 1:**
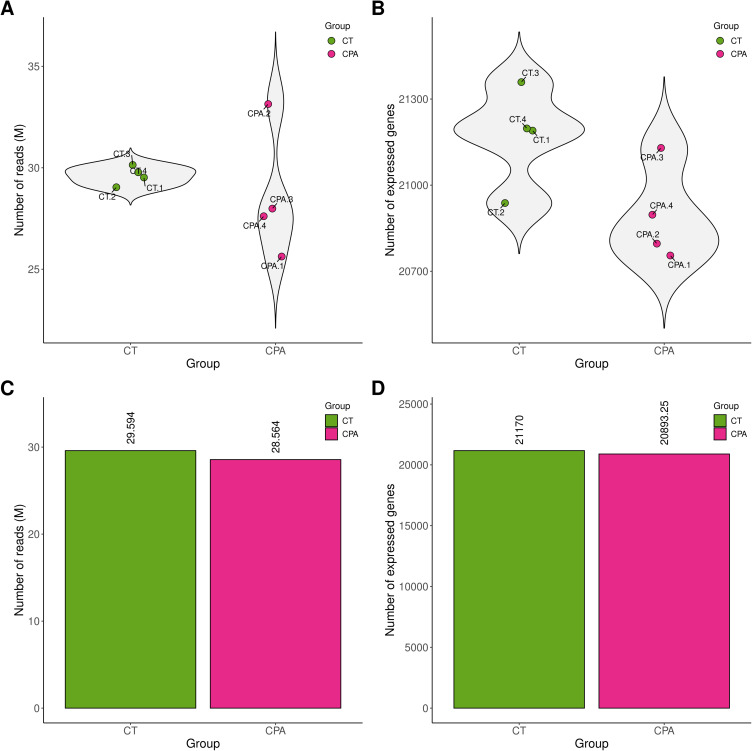
The distribution of the number of reads (**A** and **C**) and the number of expressed genes (**B** and **D**) within the ovaries of control (CT) and CPA-treated rats.

**Figure 2:**
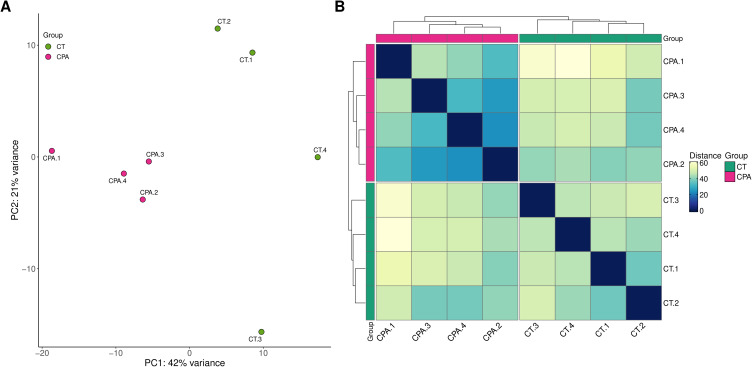
Graphical representation of the first (PC1) and second (PC2) principal components (PCA, panel (**A**) and samples distance matrix (panel (**B**) affecting transcriptome expression pattern of ovaries in control (CT) and CPA-treated rats.

The sequencing data generated in this study have been deposited in the BioProject database under accession number PRJNA640997. After removal of adapter sequences and low-quality reads, each sample contained between 32.3 and 40.9 million high-quality reads, which were subsequently aligned to the Ensembl rat genome (mRatBN7.2; Ensembl release 107). On average, 94.6% of reads mapped uniquely. Across all samples from tumor-bearing rats, the total number of expressed genes ranged from 20751 to 21358 (Table S2). The distribution of read counts (Figure 1A–C) and expressed genes (Figure 1B–D) within the control and CPA-treated groups was visualized. Both Principal Component Analysis (PCA; Figure 2A) and distance matrix analysis (Figure 2B) indicated a high degree of similarity among biological replicates within each experimental group.

**Figure 3:**
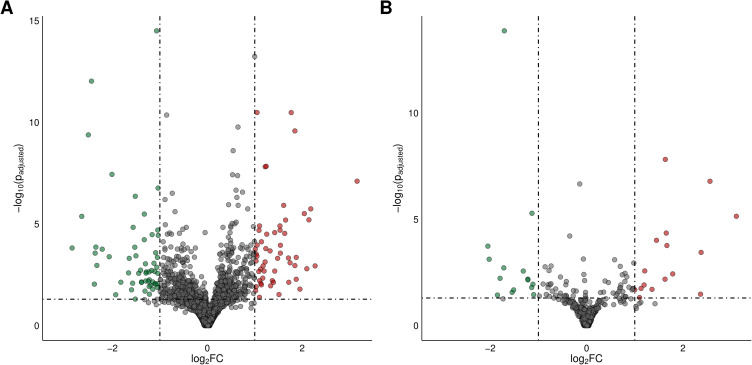
The volcano plot presenting differentially expressed genes (DEGs; P-adjusted ≤ 0.05 and log2FC ≥ 1.0 or log2FC ≤ −1.0; panel (**A**) and lncRNAs (DELs; P-adjusted ≤ 0.05 and log2FC ≥ 1.0 or log2FC ≤ −1.0; panel (**B**) identified in the ovaries of CPA-treated rats vs untreated control rats. DEGs and DELs are represented by multicolored circles, where red color depicts up-regulated DEGs/DELs and green color represents down-regulated DEGs/DELs. Gray circles represent genes or lncRNAs with unchanged expression.

**Figure 4:**
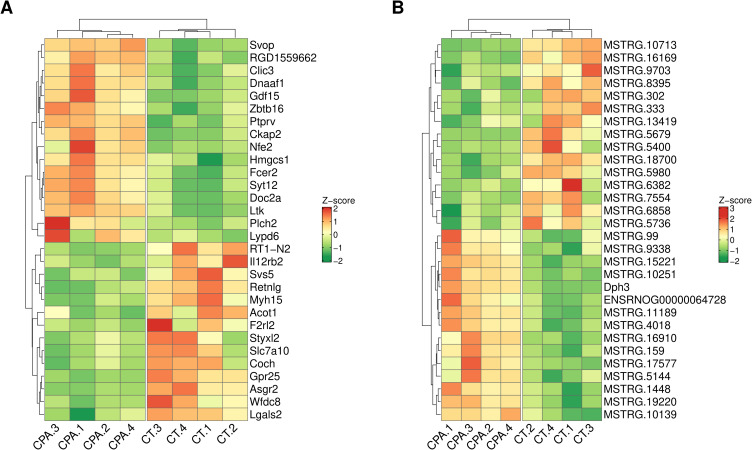
Heatmap of the top 30 differentially expressed genes (DEGs; P-adjusted ≤ 0.05 and log2FC ≥ 1.0 or log2FC ≤ −1.0; panel (**A**) and lncRNAs (DELs; P-adjusted ≤ 0.05 and log2FC ≥ 1.0 or log2FC ≤ −1.0; panel (**B**) identified in the ovaries of CPA-treated rats vs untreated rats. The expression values are presented as a Z-score calculated from raw counts for each sample. The color scale of the heatmap shows the expression level of transcripts: red blocks represent up- and green blocks represent down-regulated DEGs or DELs.

A total of 112 differentially expressed genes (DEGs) were identified in this study (Table S3). Of these, 57 DEGs were down-regulated and 55 were up-regulated in the ovaries of CPA-treated rats compared to the control group. The distribution of DEGs (P-adjusted < 0.05, |log2FC| ≥ 1.0) in the ovaries of tumor-bearing rats following CPA treatment is presented in Figure 3A. Expression profiles of the top 30 up-regulated and down-regulated DEGs (ie, those with the highest and lowest log2FC values) are illustrated in Figure 4A. The log2FC values for DEGs ranged from −2.85 (Il12rb2, interleukin 12 receptor subunit beta 2) to 3.16 (Svop, SV2 related protein 2) (Table S3).

### Functional Characterization of the Identified DEGs

**Figure 5:**
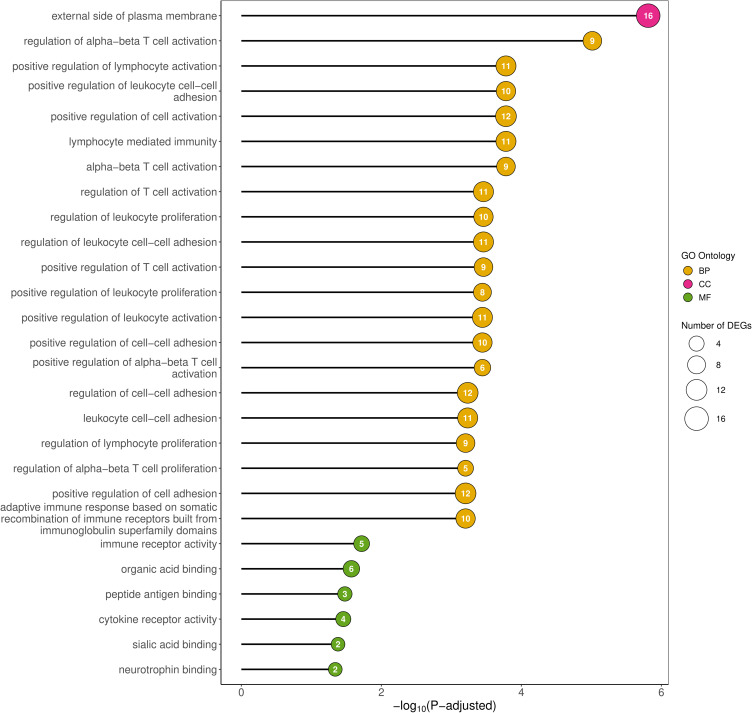
Gene Ontology (GO) analysis of DEGs identified in the ovaries of rats treated with CPA. The x-axis represents statistical significance of the GO enrichment analysis, while the y-axis denotes the name of a particular GO term. The size of the circle mirrors the number of DEGs assigned to the term, and the circle color indicates GO category.

To evaluate the potential functional relevance of the identified DEGs in the ovaries of CPA-treated rats relative to controls, the genes were categorized into three principal groups according to the Gene Ontology (GO) database: biological processes (BP), cellular components (CC), molecular function (MF). One hundred five out of 112 DEGs were ascribed to 103 GO terms (P-adjusted < 0.05) including 96 terms within BP, 1 term within CC and 6 terms within MF categories (Figure 5; Table S4). Within the BP category, DEGs were predominantly enriched in pathways associated with immune cell activation, adhesion, differentiation, and proliferation. In the CC category, DEGs were primarily mapped to the external side of the plasma membrane. Within the MF category, DEGs were largely associated with immune/cytokine receptor activity and binding of peptide antigens and neurotrophins (Table S4).

**Figure 6:**
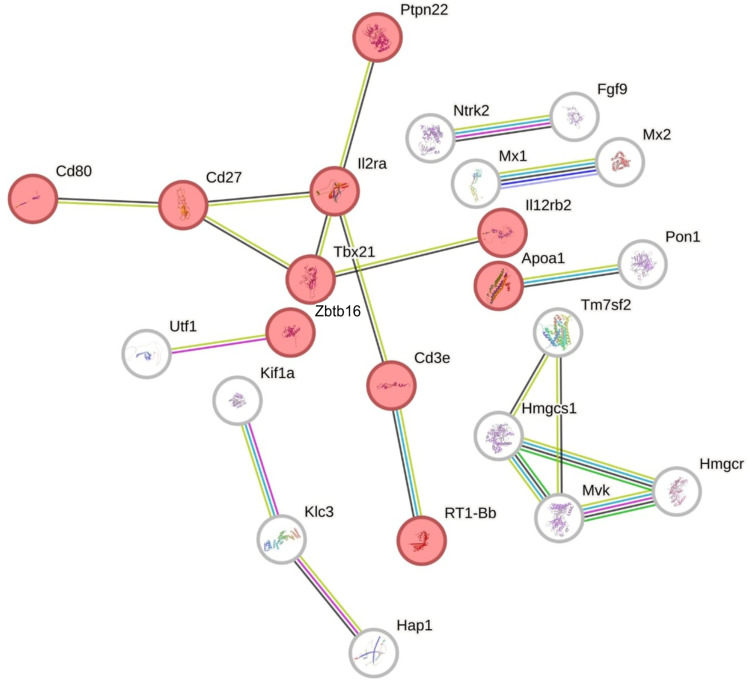
Interaction network of differentially expressed genes (DEGs) identified in the ovaries collected from CPA-treated rats. The network was generated by STRING (confidence score: 0.7; Enrichment P-value: 1.12×10^−8^ and |log2 fold change| ≥1.0). The nodes marked in red color belong to the GO regulation of immune system process term (GO:0002682).

Functional classification of the identified DEGs was further performed with the use of STRING tool (v12.0). The analysis generated a gene interaction network consisting of 103 nodes and 19 edges (Figure 6), with a protein–protein interaction (PPI) enrichment P-value of 1.12×10^−8^. Nodes that lacked any interactions were removed from the network. The constructed network encompasses genes associated with proliferation and activation of immune cells (Cd80, Cd27, Cd3e, Il2ra, Il12rb2, Ptpn22, Tbx21, Zbtb16 and RT1-Bb) and metabolism of steroids (Apoa1, Pon1, Tm7sf2, Hmgcs1, Hmgcr, Mvk). Within the analyzed interactions we also identified a group of DEGs associated with response to chemicals (eg, Hap1, Ntrk2, Fgf9, Mx1, Tbx21) (Figure 6, Table S4).

**Figure 7:**
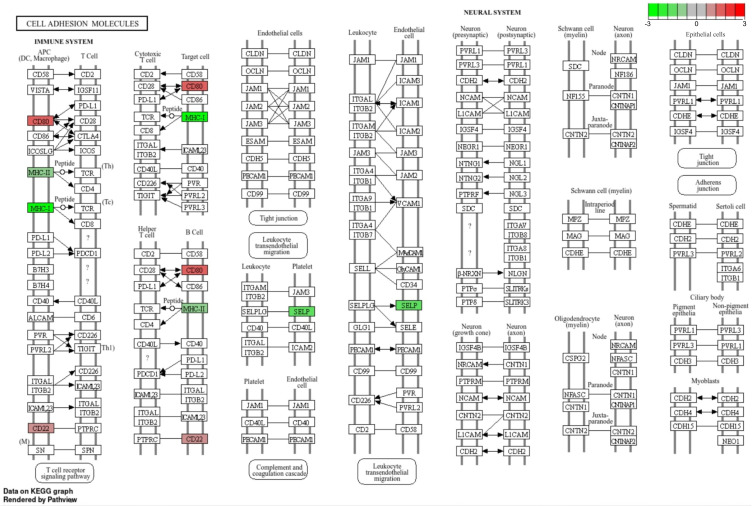
The KEGG cell adhesion molecules pathway comprising DEGs identified by RNA-Seq in the ovaries of CPA-treated rats. Red and green blocks represent up- and down-regulated DEGs, respectively. Question marks (?) indicate uncertain or not fully characterized interactions between molecules.

The KEGG database was utilized to categorize the identified DEGs (P-adjusted < 0.05) based on their functional roles. The analysis yielded 16 pathways (Table S5) involved mostly in various immune cell functions (Table S5). Moreover, two genes with expression up-regulated by CPA (CD80, CD22) and three genes with expression down-regulated (MHC-I, MHC-II, SELP) were identified within the “cell adhesion molecules” pathway (Figure 7, Table S5).

### Impact of CPA on the Ovarian lncRNA Expression Profile in Rats

**Figure 8:**
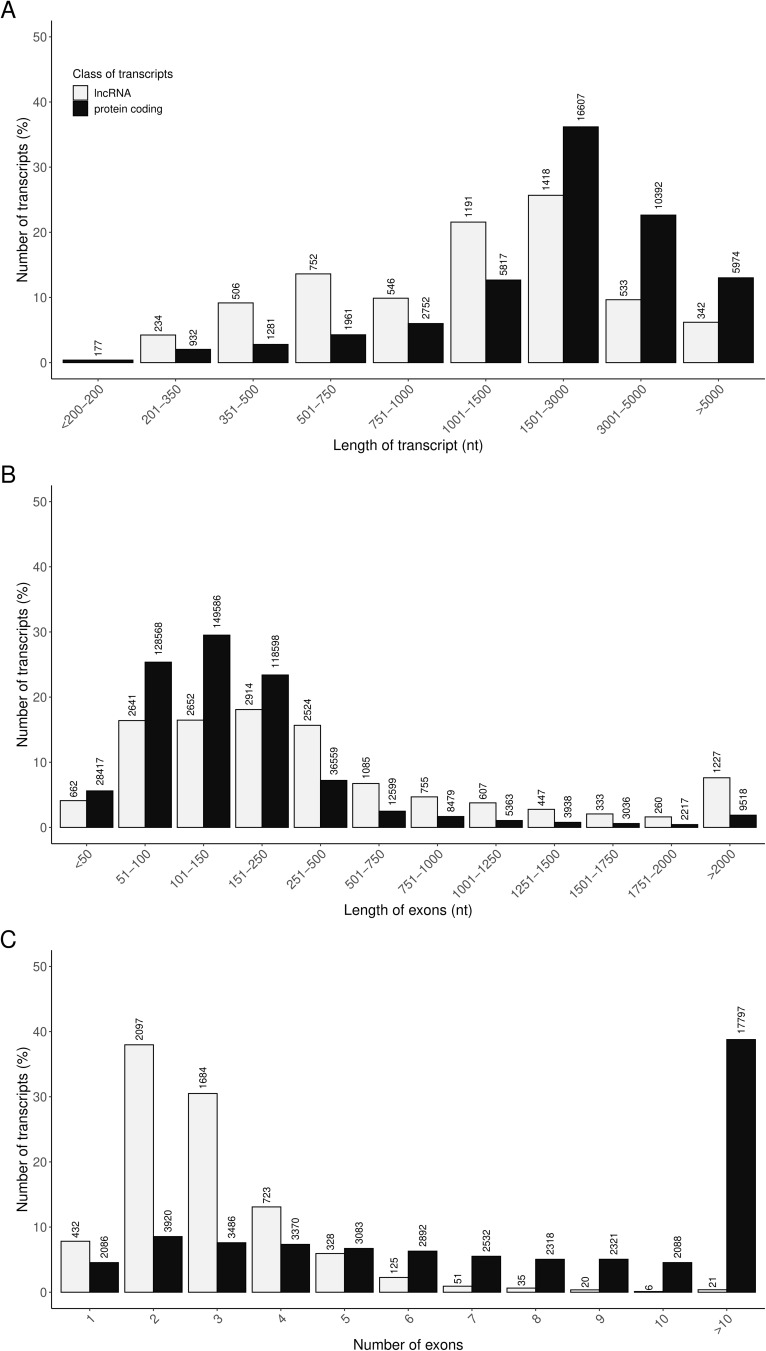
The comparison of the identified ovarian lncRNAs and protein coding transcripts according to their: (**A**) transcript length, (**B**) exon length and (**C**) exon number.

A custom multi-step pipeline was employed to distinguish lncRNAs from the full set of assembled transcripts. In total, 3888 genes were classified as lncRNAs, comprising 2488 previously annotated in the Ensembl database and 1400 novel lncRNAs. Comparative analyses of transcript length, exon length, and exon number were performed between the identified lncRNAs (5522 transcripts) and mRNAs (45893 transcripts; Table S6, Figure 8). The majority of lncRNAs and their exons had lengths ranging from 500 to 3000 nt and 50 to 500 nt, respectively (Figure 8A and B). The majority of lncRNAs consisted of 2–3 exons (Figure 8C). Moreover, the mean length of lncRNAs was shorter than that of protein coding transcripts (Table S6), and the average exon length of lncRNAs was longer than that of protein coding (Table S6). In addition, the mean exon number of lncRNAs per transcript was lower than the mean exon number of protein coding transcripts (Table S6).

**Figure 9:**
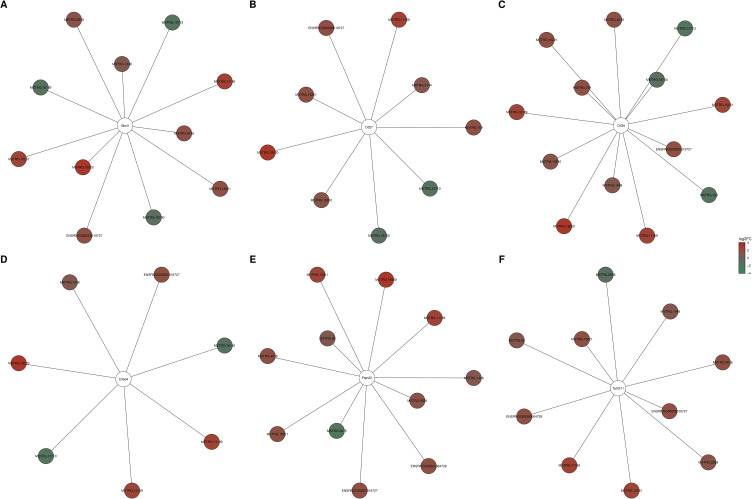
DELs and DEGs co-expression network analysis. Top positive and negative correlations of DELs with six selected DEGs (**A**) Bbc3, (**B**) Cd27, (**C**) Cd3e, (**D**) Cited4, (**E**) Ptpn22 and (**F**) Tp53i11) are visualized (Pearson correlation coefficient R2≥0.99). Red and green nodes represent the respective significantly upregulated and downregulated DELs in the ovaries of tumor-bearing rats treated with CPA.

We identified 31 DELs (|log2FC| ≥ 1 and padj⩽0.05) (Table S7) in the examined ovaries. The log2FC values for these DELs ranged from −2.05 (MSTRG.13419) to 3.11 (MSTRG.19220). Among them, 16 DELs were up-regulated, and 15 DELs were down-regulated by CPA. The distribution of DELs (P-adjusted < 0.05, |log2FC| ≥ 1.0) in the ovaries of tumor-bearing rats following CPA treatment is presented in Figure 3B. Expression profiles of the top 30 up-regulated and down-regulated DELs (ie, those with the highest and lowest log2FC values) are illustrated in Figure 4B. *In silico* analysis revealed a total of 239 negative and 446 positive correlations between the identified DELs and DEGs (Table S8). To investigate the potential functional roles of these lncRNAs in the ovarian response to CPA, the target genes for DELs were predicted. No *cis*-regulated DEGs were identified; however, all 31 DELs were predicted to potentially *trans*-regulate target DEGs (Table S9). The number of target DEGs for a given DEL ranged from 1 to 59. The negatively co-expressed DEGs were enriched in 87 GO terms (69 within BP, 5 within CC and 13 within MF) including “reproductive structure development” (GO:0048608), “regulation of developmental growth” (GO:0048638) and “enzyme inhibitor activity” (GO:0004857) (Table S10). These DEGs involved: Plac8, Lyc2, Serpina5 and C3 (Table S10). The positively co-expressed DEGs were enriched in 87 GO terms (84 within BP, 1 within CC and 4 within MF). Several of these DEGs were associated with „positive regulation of cell activation” (GO:0050867), “regulation of cell-cell adhesion” (GO:0022407) and „leukocyte proliferation” (GO:0070661) (Table S11). This group of trans-target DEGs included: Cd27, Cd3e, Cd80, Ptpn22, Selp, Zbtb16, Il2ra, Il12rb2, Tbx21, RT1-Bb and Ccr2 (Table S11). The strongest positive and negative correlations between DELs and six selected DEGs (Bbc3, Tp53i11, Cited4, Cd3e, Cd27, Ptpn22) are presented in Figure 9A–F.

### Validation of RNA-Seq results Using Quantitative Real-Time PCR

To confirm the RNA-Seq results, two DEGs were selected for real-time PCR analysis: Bbc3 (log2FC: 1.05) and Il12rb2 (log2FC: −2.85). The expression levels of these DEGs, as measured by real-time PCR, were consistent with the RNA-Seq findings (Figure S1).

### Impact of CPA on the Ovarian Proteome in Rats

A DIGE-based proteomic approach was used to identify differentially expressed protein spots (DEPSs) in the ovaries of tumor-bearing rats following CPA treatment. Across all gels, 672 DEPSs were detected, of which 596 were successfully matched between CPA-treated and control samples. Figure S2 shows a representative gel image. The abundance of 56 DEPSs was found to differ significantly (P < 0.05; |fold change| > 1.5) between the examined groups. Twenty two DEPSs were identified with the use of MALDI TOF/TOF MS. The identified proteins include: vimentin (Vim), annexin A5 (ANXA5), prohibitin (Phb), heat shock cognate 71 kDa protein (Hsp7c), mitochondrial aldehyde dehydrogenase (Aldh2), fructose-bisphosphate aldolase A (Aldoa), phosphoglycerate kinase 1 (PGK1), calretinin (CALB2), 60 kDa heat shock protein, mitochondrial (CH60), tropomyosin alpha-3 chain (TPM3) and glucose-6-phosphate 1-dehydrogenase (G6PD) (Table 1).

**Table 1:** Differentially Expressed Proteins Identified in the Ovaries of Mammary Tumor-Bearing Rats Treated with Cyclophosphamide vs Control Rats

	Identified Proteins	MASCOT Protein Score	Sequence Coverage [%]	Number of Peptides	Fold Change	Accession Number
1	60 kDa heat shock protein, mitochondrial (CH60)	228	29	11	−2.6	gi ǀ206597443
2	Calretinin (CALB2)	113	14	4	−2.2	gi ǀ16758892
3	10 kDa heat shock protein, mitochondrial (CH10)	163	10	3	−2.1	gi ǀ6981052
4	Fructose-bisphosphate aldolase A (ALDOA)	108	16	4	−2.0	gi ǀ408772019
5	Protein disulfide-isomerase (PDIA1)	253	22	9	−2.0	gi ǀ129731
6	Glyceraldehyde-3-phosphate dehydrogenase (G3P)	82	4	1	−2.0	gi ǀ8393418
7	Serum albumin (ALBU)	332	29	14	−2.0	gi ǀ158138568
8	Vimentin (VIM)	287	26	12	−1.7	gi ǀ38197662
9	Glucose-6-phosphate 1-dehydrogenase (G6PD)	69	12	5	−1.7	gi ǀ8393381
10	Aldehyde dehydrogenase (AL1A1)	89	18	6	−1.7	gi ǀ14192935
11	Tropomyosin alpha-3 chain (TPM3)	722	49	22	−1.6	gi ǀ669633260
12	Phosphoglycerate kinase 1 (PGK1)	52	3	1	−1.6	gi ǀ56585024
13	Prohibitin (PHB)	83	21	5	−1.5	gi ǀ13937353
14	Tropomyosin beta chain (TPM2)	103	16	6	−1.4	gi ǀ669033265
15	Heat shock cognate 71 kDa protein (HSP7C)	118	16	7	1.6	gi ǀ13242237
16	Annexin A5 (ANXA5)	137	32	8	1.7	gi ǀ1937369594
17	Ferritin light chain 1 (FRIL1)	217	37	2	1.8	gi ǀ204133
18	Aldehyde dehydrogenase, mitochondrial (ALDH2)	263	16	8	2.4	gi ǀ1820958497
19	Rho family-interacting cell polarization regulator 2 (RIPR2)	181	5	2	2.4	gi ǀ110625641
20	Ferritin heavy chain (FRIH)	198	32	2	2.5	gi ǀ6978859
21	Actin, cytoplasmic 1 (ACTB)	105	35	1	2.5	gi ǀ13592133
22	Superoxide dismutase Cu-Zn (SODC)	339	47	7	2.7	gi ǀ8394328

**Figure 10:**
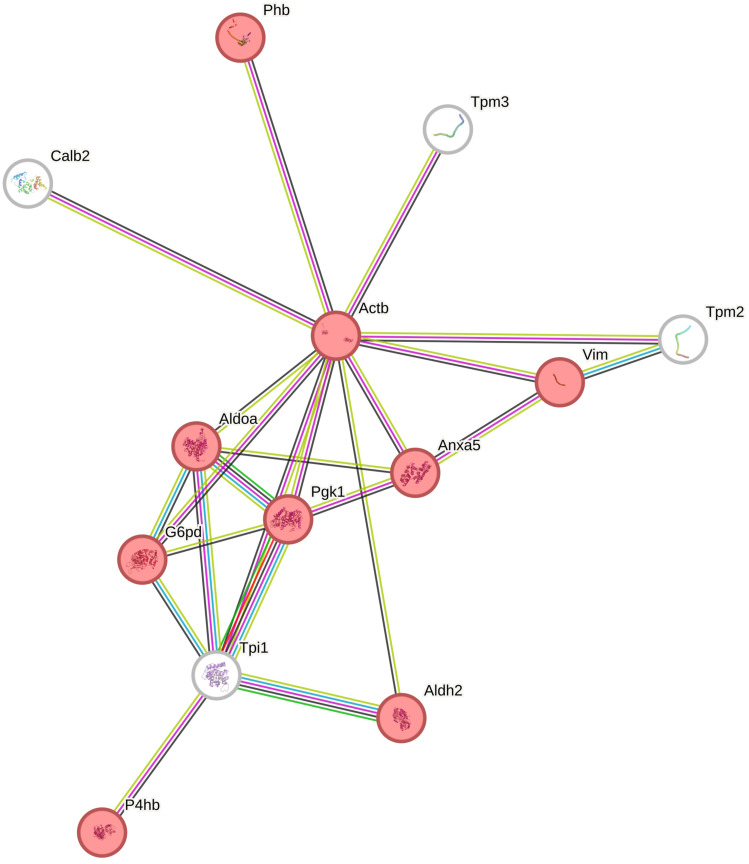
Interaction network of differentially expressed proteins (DEPSs) identified in the ovaries collected from CPA-treated rats. The network was generated by STRING (confidence score: 0.7; Enrichment P-value: 4.16×10^−12^ and |log2 fold change| ≥1.0). The nodes marked in red color belong to the GO response to stress term (GO:0006950).

Functional classification of the identified differentially expressed proteins was further carried out using the STRING tool. The analysis generated a protein interaction network consisting of 13 nodes and 23 edges (Figure 10), with a protein–protein interaction (PPI) enrichment P-value of 4.16×10^−12^. Nodes without any interactions were removed from the network. The resulting network included proteins associated with “response to stress” and “cellular response to chemical stimulus” (Phb, Actb, Vim, Aldoa, Anxa5, Pgk1, G6pd, Aldh2 and P4hb; Figure 10).

## Discussion

We showed previously that CPA exposure leads to a decline in primordial and primary follicle numbers in tumor-bearing rat ovaries.^4^ Moreover, we found that granulosal apoptosis was greater in the ovaries harvested from CPA-treated cancer rats than in controls.^4^ These data are in agreement with reports demonstrating CPA-induced ovarian toxicity in women and animals.^3^,^20^ In the present study, we investigated the molecular mechanisms underlying CPA’s effects in the ovaries of rats examined in our previous experiment.^4^ Using transcriptomic and proteomic techniques, we identified 112 DEGs, 31 DELs and 22 DEPSs in the ovaries of CPA-treated animals. Most of these molecules were enriched in Gene Ontology categories associated with activation, adhesion, differentiation and proliferation of immune cells as well as with steroid metabolism and responses to chemicals.

The molecular actions of CPA underlying its toxicity in the ovary were shown to involve three major mechanisms: 1/ direct toxic effect on primordial follicles, with apoptosis as a main cause of follicular depletion, 2/ an indirect destructive effect through ovarian stromal tissue and 3/ an indirect action on primordial follicles via over-recruitment causing “burn-out” effect of primordial follicle reserve.^3^ All these actions may result in reduced fertility and reproductive outcomes in chemotherapy-treated females. Our previous study further demonstrated that CPA decreased primordial and primary follicle counts in tumor-bearing rats by inducing apoptosis.^4^ Not many apoptosis-related genes or proteins were found to be affected by CPA in the current study. We demonstrated, however, that CPA increased the expression of Bbc3 (PUMA) and Tp53i11 genes (Table S3). PUMA has been identified as a crucial apoptotic trigger for the depletion of primordial follicles induced by CPA in mice.^21^ The effects of Tp53i11 on apoptosis are usually positive, but its significant involvement in angiogenesis was also recently discovered in the tumor microenvironment.^22^ Moreover, in the present study, CPA decreased expression of CITED4 gene (Table S3). Knockdown of CITED4 in ovine granulosa cells increased apoptosis and expression of apoptosis-related genes.^23^ In addition, CITED4 was identified as a key transcription factor involved in ovulation in mice.^24^ These data suggest that CPA in our tumor-bearing rat model depleted primordial and primary follicles through apoptosis pathways.

Although CPA is best known as an antineoplastic therapy, it is also used as an immunosuppressive drug for autoimmune diseases.^25^,^26^ Interestingly, in the current study, the most of GO terms associated with the identified DEGs were related to regulation of immune cells (Figure 5). Also, GO analysis of the trans-target genes predicted to be regulated by the DELs identified in this study, indicated many processes important for immune cell functions. Among these DEGs we found the following genes: Il12rb2, Il2ra, Cd27, Cd80, Cd3e, Ptpn22, Zbtb16, Tbx21 and RT1-Bb (Table S9). Moreover, within the group of CPA-affected ovarian proteins we identified RIPR2 and annexin A5 reported to be related to immunosuppression.^27^,^28^ Il12rb2 gene encoding subunit β of interleukin 12 receptor – the gene most strongly downregulated by CPA in the current study – is predominantly expressed in NK and T cells.^29^ This receptor is known to activate proliferation of NK/T cells and differentiation of T helper cells. Moreover, we demonstrated that CPA inhibited expression of Il2ra (CD25) gene which is present in T regulatory (T-reg) cells that play a crucial role in preventing autoimmune diseases and tumor-induced tolerance.^30^ These data support previously published results on immunosuppressive action of CPA, involving selective and rapid decrease in the number of T-reg cells in cancer models^31^ and cancer patients.^32^ Previous studies suggested that immune cell disfunctions are associated with ovarian failure.^33^,^34^ Immune-mediated ovarian damage may be caused by several mechanisms including a reduction in the number of suppressor/cytotoxic lymphocytes and a decrease in both the number and activity of NK cells.^35^ It seems that the crucial mechanism of CPA action during follicular depletion may be related to the CPA-induced responses of immune cells present within the ovaries. An immunosuppressive action of CPA may explain, at least in part, the adverse effects of this drug on the number of follicles demonstrated in the same animal model employed in our previous study.^4^

Recent reports have shown that ferroptosis, a newly discovered intracellular mechanism of cell death, may be responsible for the ovarian toxicity induced by chemotherapeutics.^7^,^36^ Ferroptosis represents an iron-dependent, non-apoptotic cell death pathway defined by uncontrolled lipid peroxidation and oxidative damage, ultimately resulting in plasma membrane rupture and the release of intracellular contents.^37^,^38^ Ferroptosis results from pro-oxidant–antioxidant imbalance, and its highly complex regulation remains under investigation. In the current study, CPA decreased the expression of Slc7a10 gene (Figure 4, Table S3) and increased the abundance of ferritin heavy chain, ferritin light chain 1 and superoxide dismutase Cu-Zn proteins (Table 1) – all of which are known to be involved in ferroptosis. Slc7a10 and Slc7a11 transporters are anti-ferroptotic proteins, whose inhibition can result in the accumulation of iron-dependent lipid peroxidation, thereby triggering ferroptotic cell death.^37^,^39^ Ferritin, in turn, is a cytosolic iron storage protein, and its degradation by lysosomes results in increasing free iron levels and induction of ferroptosis.^38^ Like our results, Dai and co-workers reported that CPA increased ferritin in granulosa cells both in vivo and in vitro.^7^ Decreased activity of antioxidant enzymes such as superoxide dismutase are also required to cause ferroptosis.^40^ Although the above data demonstrate the effects of CPA on the expression of genes/proteins related to ferroptosis, a direct effect of CPA on the occurrence of ferroptosis was not examined in the current study. Nonetheless, the data highlight the potential of ferroptosis in contributing to ovarian toxicity from cancer chemotherapy, paving the way for developing targeted strategies for ovarian protection.

Chemotherapy is central to cancer treatment and this makes ovarian damage inevitable in female cancer patients. Our present study demonstrated CPA-induced molecular alterations in the ovaries of tumor-bearing rats, suggesting that CPA may exert immunosuppressive effects (*Il12rb2, Il2ra, Cd27, Cd80*), promote apoptosis (*Bbc3, Tp53i11, CITED4*), and trigger ferroptosis-related processes (*Slc7a10*, ferritin, superoxide dismutase), all of which may contribute to ovarian dysfunction. Given the high sensitivity of ovarian tissue to hormonal and cytotoxic insults, clarifying mechanisms underlying chemotherapy-induced injury is crucial for fertility preservation strategies. The obtained transcriptomic and proteomic data indicate key pathways potentially involved in CPA-mediated ovarian damage. Our previous findings showed that tamoxifen may prevent the depletion of the ovarian follicular reserve in CPA-treated rats with mammary tumors^4^ and that its mechanism of action may include the effects on ovarian steroidogenesis and primordial follicle activation or arrest.^41^,^42^ Therefore, tamoxifen could represent a promising tool for mitigating chemotherapy-related ovarian toxicity. Scientists are still looking for ways to preserve fertility during and after cancer therapy^43^ and consequently improve the quality of life for cancer patients.

## Conclusions

Cyclophosphamide, widely used in the treatment of cancers (including breast cancer) and autoimmune diseases, exerts significant gonadotoxic effects leading to ovarian reserve depletion and infertility. In this study, the ovarian molecules affected by CPA exposure were associated with pathways involved in immune activation, cell adhesion, proliferation, ferroptosis, steroid metabolism, and stress response. These findings highlight multiple molecular mechanisms through which CPA may impair ovarian function and provide a foundation for future research aimed at developing fertility-preserving strategies for women undergoing chemotherapy.

## Data Availability

The sequencing data from the current study were submitted to the BioProject database under accession number PRJNA640997.
